# Droplet fluidics for time-dependent analysis of barrier permeability in an epithelial barrier on chip system†

**DOI:** 10.1039/d3ra00470h

**Published:** 2023-05-11

**Authors:** Joao Fernandes, Nikita Karra, Emily J. Swindle, Hywel Morgan

**Affiliations:** a Electronics and Computer Science, Faculty of Physical Sciences and Engineering, University of Southampton UK hm@ecs.soton.ac.uk; b Clinical and Experimental Sciences, Faculty of Medicine, University of Southampton UK; c Institute for Life Sciences, University of Southampton UK; d NIHR Southampton Biomedical Research Centre, University Hospital Southampton UK

## Abstract

A droplet generator has been developed that interfaces with a barrier-on-chip platform for temporal analyte compartmentalisation and analysis. Droplets are generated every 20 minutes in 8 separate parallel microchannels, with an average droplet volume of 9.47 ± 0.6 μL, allowing simultaneous analysis of 8 different experiments. The device was tested using an epithelial barrier model by monitoring the diffusion of a fluorescent high molecular weight dextran molecule. The epithelial barrier was perturbed using detergent leading to a peak at 3–4 hours, correlating with simulations. For the untreated (control) a constant, very low level of dextran diffusion was observed. The epithelial cell barrier properties were also continuously measured using electrical impedance spectroscopy to extract an equivalent trans epithelial resistance.

## Abstract 2

A droplet microfluidics platform for time-dependent analysis of epithelial cell barrier permeability used in conjunction with an organ-on-chip system.

## Graphical abstract

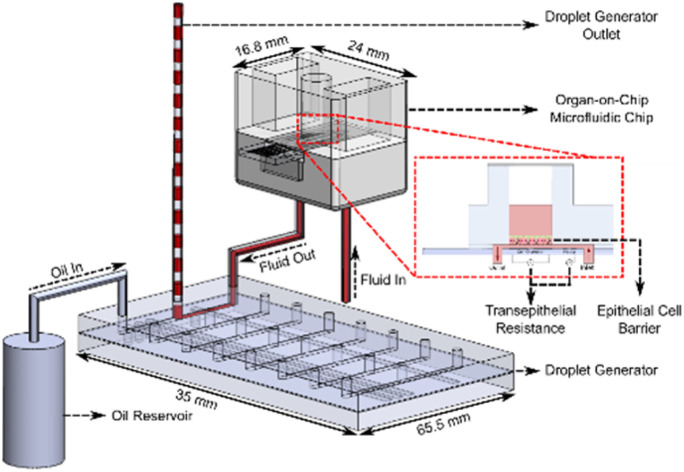

## Introduction

Molecular permeability and protein expression measurements in Organ on Chip (OoC) systems are usually determined as end point values, where the extracellular medium is collected at the end of an experiment and analysed using classical methods.^1–4^ While measurement of Trans Epithelial Electrical Resistance (TEER) is the most common method for determining epithelial barrier integrity, this metric is not capable of determining the range of analytes/biomarkers released by cells. It also only measures one aspect of barrier integrity, ionic permeability and does not give an indication of the macromolecular permeability of the epithelial barrier. Furthermore, dynamic, time-dependent analysis of protein secretions would provide much greater insight into cell behaviour. Multiplex analysis of proteins is generally performed using bead-based (Luminex beads or AlphaLisa) or electrochemical assays,^5–11^ with bead-based assays performed as endpoint measurements.^12,13^ Integration of antibody/aptamer covered electrodes for time-dependent analysis of proteins using electrochemical assays has been reported.^14^ For example, Shin *et al.*,^15^ developed an electrochemical sensor for analysis of albumin and GST-α. The sensor was integrated into a liver-on-chip platform, enabling automatic and continuous monitoring of cell-secreted biomarkers from human liver organoids for 7 days. Electrochemical sensors can also be externally connected to OoC devices.^16,17^ Lee *et al.*,^18^ described a sensor for simultaneous detection of troponin, creatine kinase MB (CK-MB) and human epidermal growth factor receptor 2 (HER-2) from a dual-organ platform in which cardiac and breast cancer tissue was cultured. Each electrode was covered with antigen-specific aptamers for analyte detection. The sensor was used in a dual-organ platform, with measurements of analyte concentration performed every 2 days, during 5 days of culture.

Hu *et al.*,^19^ described a droplet-microfluidic device that sampled secretions from primary adipose tissue explants with in-line quantification of glycerol. Adams *et al.*,^20^ develop a microfluidic bioreactor containing HepG2 cells. The authors measured glucose by removing the bioreactor from the incubator, adding reagents and interfacing with a droplet-microfluidic system.

OoC devices can also be interfaced with classical fraction collectors to intermittently collect small amounts of cell media into separate reservoirs, allowing post-analysis of eluate stored in each reservoir. Blume *et al.*,^21^ described a platform that used standard Transwells with basolateral microfluidic flow to capture media from primary human bronchial epithelial cells. The platform was coupled to an automated fraction collector for time-dependent analysis of interleukin-8 release over a period of 24 hours. More recently, Nawroth *et al.*,^22^ reported the use of a custom-made fraction collector for temporal analysis of IFN-I1, CXCL10 and IL-6 secretions following HRV infection of co-cultures of primary airway epithelial cells with endothelial cells (human microvasculature or human umbilical vein cells). Basolateral media was dispensed into a 96 well plate in two-hour intervals over 72 hours, and an increase of analyte concentration was visible in infected cultures.

Molecular permeability in organ-on-chip devices is usually measured by monitoring transport of large molecular weight fluorescent markers. This can be performed on-chip if the device is optically clear and fits in a fluorescent microscope stage. Chrobak *et al.*,^23^ described the formation and maturation of microvascular tubes inside a PDMS device using Alexa-Fluor labelled 3 kDa dextran and BSA. Fluorescent images of the cell culture were captured every 20 minutes inside an environmental chamber at 37 °C and treatment of the tubes with histamine resulted in changes to barrier permeability. Villenave *et al.*,^24^ described a human gut epithelial molecular permeability assay using an Emulate organ-on-chip platform, in which inulin-FITC was perfused in the apical channel at 60 μL h^−1^ for 24 hours. Liquid collected at the basolateral and apical outlets of the device were analysed and showed an immediate decrease of permeability following cell seeding.

Droplet microfluidics provides an alternative approach for analyte collection and analysis and has been used for continuous sampling and analysis of tissue, for example to monitor biomarkers in real-time *in vivo*.^25,26^ Evans *et al.*,^27^ described a portable droplet microfluidic device capable of collecting samples (*ex vivo*), producing droplets, and performing a magnetic bead-based ELISA with colorimetric readout. The device detected cortisol in 10 minutes with a detection range between 3.175 and 100 ng mL^−1^. Nightingale *et al.*,^25^ demonstrated a wearable droplet collection for autonomous analysis of glucose and lactate. A microdialysis probe was connected to a peristaltic pump, encapsulating samples into nanolitre sized droplets. Assay reagents were mixed with the sample inside the droplet and measured optically. Cedilla-Alcantar *et al.*,^28^ described a pneumatic device that generated droplets for *in situ* detection of glucose, bile acids and lactate dehydrogenase from an OoC platform. Droplet generation and reagent dispensing was controlled *via* pneumatic valves and detection of each analyte was performed in 3 μL samples. The platform was directly connected to the outlet of an OoC device for hepatocyte spheroid culture. Analytes were measured once per day over the course of 4 days. Although droplet microfluidics enabled streamlining of sample collection and analysis, samples were only collected daily. The system also uses several different pneumatic lines which could limit scalability.

In this paper we describe a droplet system that allows temporal collection of secretions with down-stream analysis by conventional method. The device was designed to interface with a barrier on chip platform with epithelial cells grown at a liquid–liquid or air–liquid interface. The system was characterised by measuring the time dependent transport of fluorescent dextran through a polarised bronchial epithelial cell layer, before and after barrier disruption. Its simple design and function allows for simultaneous compartmentalisation of basolateral cell media from at least 8 microfluidic chips in parallel.

### System design

**Fig. 1:**
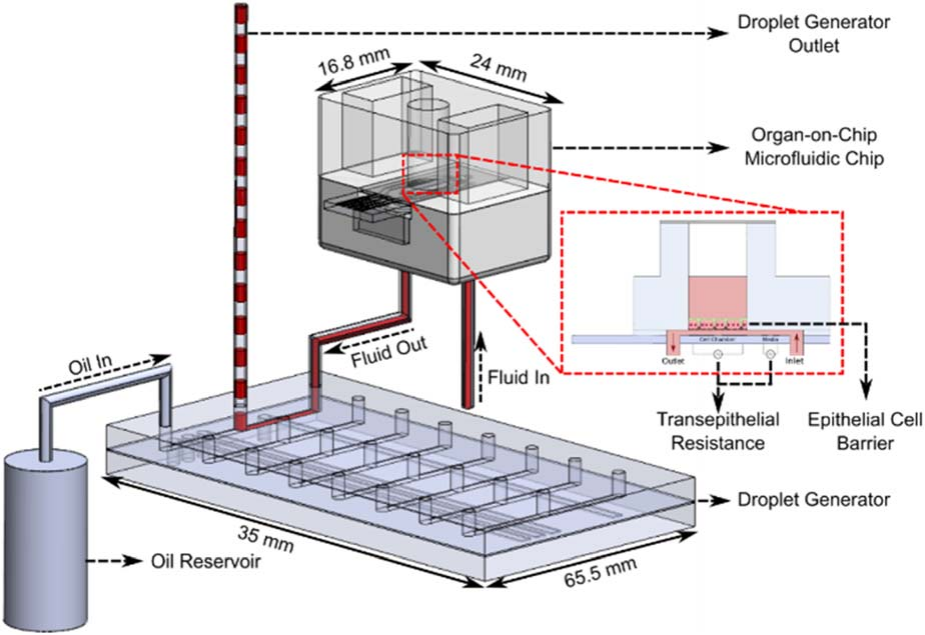
Schematic diagram of the droplet generator coupled to the OoC chip. Media flows beneath the support membrane on which epithelial cells are cultured. Trans-epithelial resistance (TEER) measurements are performed automatically during cell culture with the micro-electrodes in each microfluidic chip. The out-flow containing cell secretions is compartmentalised into separate droplets (separated by oil) at discrete time intervals. The droplets are stored in the outlet tubing for off-stream analysis.

The OoC droplet system is shown in Fig. 1. A disposable microfluidic chip consisting of two compartments is separated by a nano-porous support membrane onto which cells are cultured. The upper compartment (apical) is exposed to air or can contain fluid. The lower compartment (basolateral) has media pumped at a constant flow rate beneath the support membrane. To compartmentalise the basal flow into droplets, the output from the chip enters a T-junction where the continuous flow is segmented using oil. A manifold with PDMS microvalves controls the alternate injection of the sampled media or oil, creating droplets that are stored in a long tube. Detailed information regarding fabrication of the droplet generator is available in the ESI.†

**Fig. 2:**
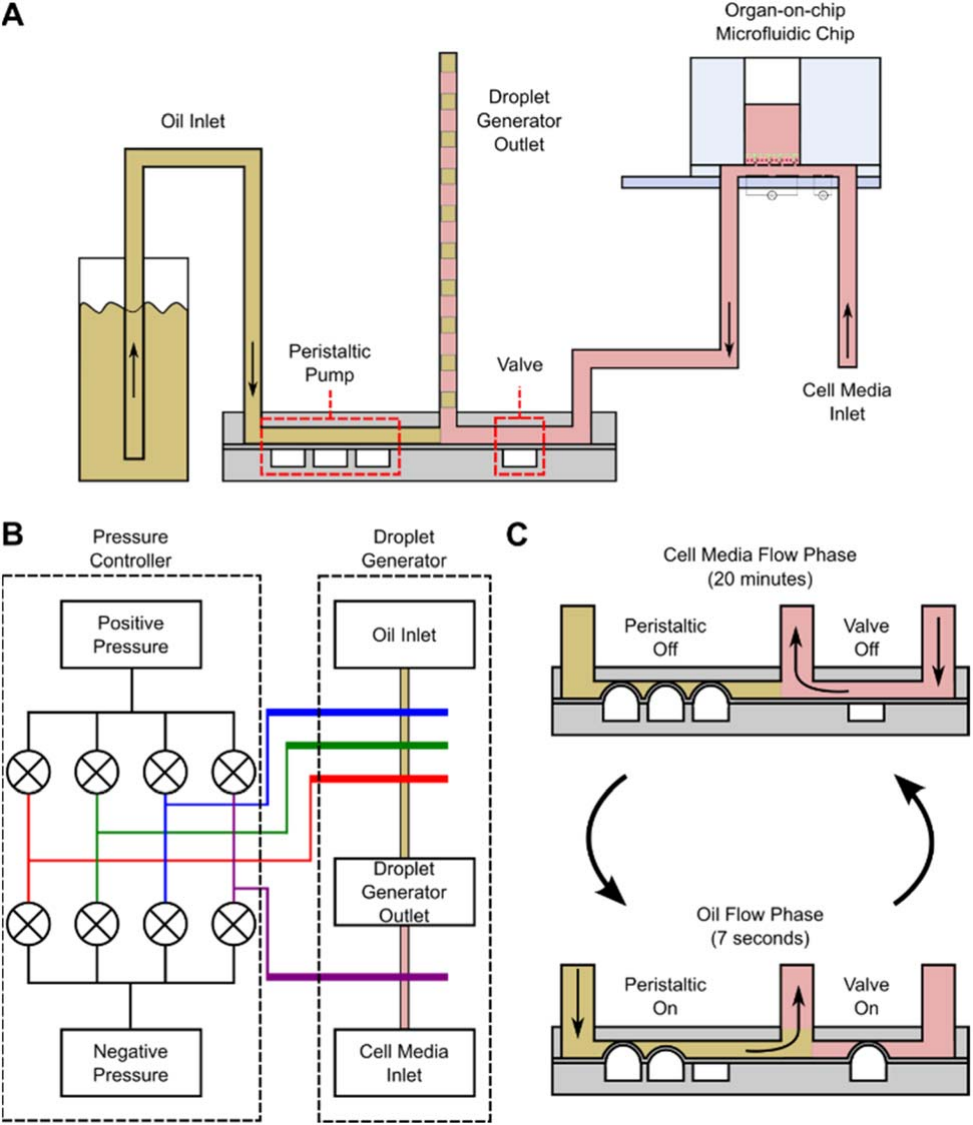
(A) Cross-section of the droplet generator showing the fluid inlets (to the oil reservoir and OoC platform) and outlets, the peristaltic pump and the PDMS valve. (B) Diagram showing the connections between the peristaltic controller and droplet generator. Each coloured line represents a different pressure connection between the 2 devices. (C) Droplet generation workflow schematic. Media flows for 20 minutes, then oil for 7 seconds. These two steps alternate for the duration of the experiment. Sequential droplets of media and oil are generated and stored at the outlet tubing with 10 μL volume each.

The oil flow is controlled *via* 3 microvalves that together create a miniature peristaltic pump. The continuous aqueous flow is provided by a syringe pump that drives liquid through the chip at 30 μL h^−1^. To create droplets this flow is momentarily interrupted using one additional microvalve (see Fig. 2). Sequential activation of the aqueous and oil phase creates droplets at the T-junction which are stored for post-analysis as shown in Fig. 1. The system has 8 microfluidic chips connected in parallel, and the droplet generator collects samples from all 8 chips. Flow between the organ-on-chip microfluidic chip and droplet generator can be affected by Taylor dispersion, with an effective diffusion of 10^−4^ m^2^ s^−1^ across the 10 cm of tubing between both devices (Fluid Out tubing in Fig. 1). The sample from the microfluidic chip takes ∼120 seconds to reach the droplet generator before being compartmentalised and the Peclet number is around of 25 000, indicating minimal Taylor dispersion.

A cross-section of the droplet generator architecture is shown in Fig. 2A, along with a diagram of the fluidic connections between the pressure controller and droplet generator (Fig. 2B), and the droplet generation workflow (Fig. 2C). The droplet generator comprises a pressure controller and droplet generator made in PDMS. The pressure controller routes positive or negative pressures (*via* solenoid valves) to the droplet generator pressure channels, actuating the PDMS microvalves. The droplet generator enables or interrupts flow of oil or cell culture media to the outlet tubing *via* these microvalves. The oil flow is generated with the peristaltic micro-pump (3 microvalves in series). The media flow is enabled/disabled by actuating the additional microvalve in the device. Assuming full microvalve actuation, the oil volume in the droplet generator is 0.45 μL per actuation whilst media from the OoC platform arrives at a constant flow rate of 30 μL h^−1^. The medium flows for 20 minutes giving a volume of 10 μL for each droplet. During this time, the peristaltic pump channels are pressurized, blocking flow of cell culture media into the oil reservoirs. To create an oil droplet in the outlet tubing, the peristaltic pump is actuated 22 times for 7 seconds, providing a volume displacement of 10 μL (0.45 μL × 22). During oil flow, the valve blocks media, preventing backflow of oil into the chips. Repeating these two steps creates a sequence of 10 μL droplets interspersed with oil. At the end of the experiment the droplets in the sample collection tubing are dispensed into micro-well plates for further analysis.

## Materials and methods

The OoC platform was fabricated as described previously.^29^ Briefly the microfluidic chips were made from PMMA bonded to a glass substrate using 50 μm double-sided adhesive tape. The microfluidic chip interfaced to a manifold using magnets and O-rings. The device was sterilised prior to use with a solution of 1 : 50 sodium hypochloride. Following decontamination, cell media was pumped through the platform, which was kept inside a humidified incubator at 37 °C with 5% CO_2_. Cell culture procedures for the human bronchial epithelial cell line (16HBE14o-) in the OoC platform are detailed in Fernandes *et al.*^29^ Cells were cultured in the system for 4 days prior to apical stimulation with Triton X-100 and fluorescein-Dextran.

The PMMA layers for the pressure controller were assembled using 50 μm double-sided adhesive tape. Each layer was machined using a laser cutter and holes for the inlet and outlet connectors were manually threaded. Solenoid valves (LFN series, The Lee Company) were bolted to the PMMA and controlled with a microcontroller connected to a laptop. Constant pressures of ±500 mBar was applied at the positive and negative inlets with two Lineup™ Series microfluidic pressure controllers (Fluigent). Pressure was controlled *via* an Arduino script and the outlets were connected to the PDMS droplet generator with PTFE tubing.

The droplet generator was made of three layers of PDMS. The top and bottom layers were cast from 3D printed Veroclear moulds, with a 1 : 10 ratio elastomer and curing agent. The middle PDMS membrane was 200 μm thick (Silex). All three layers were bonded with oxygen plasma. Before attaching to the OoC, the droplet generator channels were primed with oil (FC-40 Fluorinert Oil,) and cell media droplets (10 μL) were generated every 20 minutes over 24 hours. At the end of the experiment, the droplets were manually dispensed from the tube using a syringe pump (flowrate of 100 μL min^−1^) into a 384 well plate.

To induce epithelial barrier disruption, the polarised epithelial cells were apically stimulated with Triton X-100 (TX-100, final concentration 1% v/v), and the molecular permeability analysed by the flux of fluorescein-Dextran (FITC-Dextran) 10 kDa (final concentration 2 mg mL^−1^) from the apical to the basolateral compartment. Both solutions were diluted in minimal essential medium supplemented with 10% foetal bovine serum and 1% penicillin/streptomycin.

## Results and discussion

**Fig. 3:**
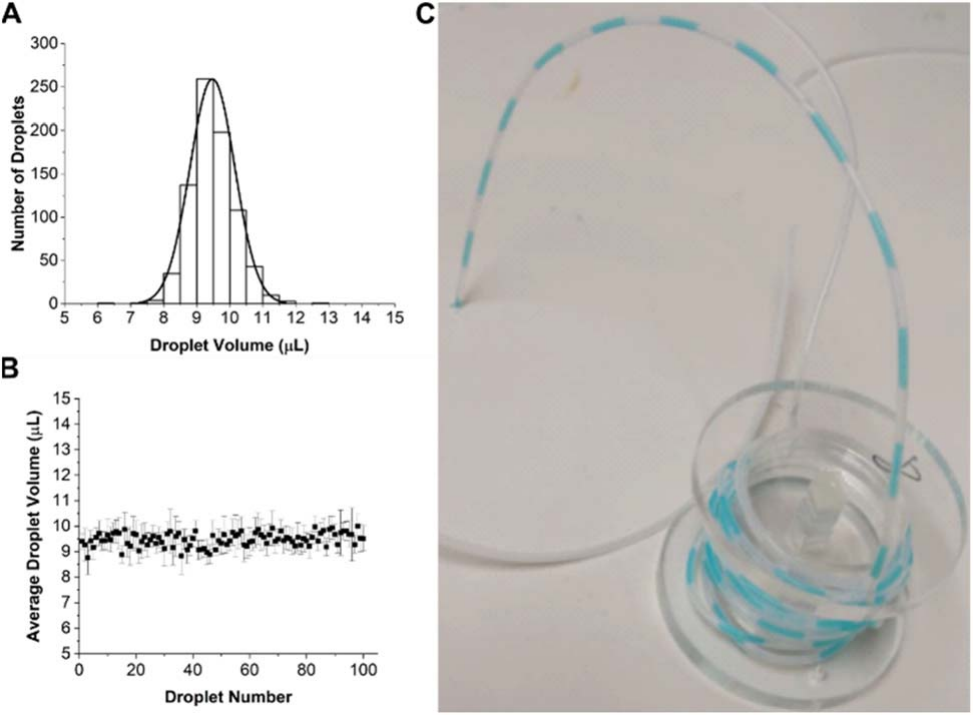
(A) Average droplet volume histogram for all 8 parallel microfluidic channels measured over 33.3 hours, generating a total of 100 droplets per channel. (B) Variation in droplet size as a function of time throughout an experiment for all channel, results are mean ± standard deviation, *n* = 8 channels. The *x*-axis indicates when a droplet is created. (C) Image showing droplets stored in tubing for off-stream analysis.

The performance of the droplet generator was first assessed using dyed water. This was pumped through the 8 chips in parallel at a flow rate of 30 μL h^−1^ for 33.3 hours, giving a total of 100 droplets, each with a volume of 10 μL. The coefficient of variation (CV) of droplets was determined by measuring the length of each segment in the tube using image analysis software (ImageJ). The distribution in droplet volume and size is shown in Fig. 3, together with an image of the droplets in the outlet tubing.

Fig. 3A shows that the droplet volume distribution is Gaussian with a mean volume of 9.47 ± 0.6 μL close to the expected value of 10 μL. Droplet stability is shown in Fig. 3B with data aggregated from all 8 channels showing a CV of 6%. The slightly lower mean volume could be due to human error in droplet volumes measurements, as their size was manually measured from a camera image.

**Fig. 4:**
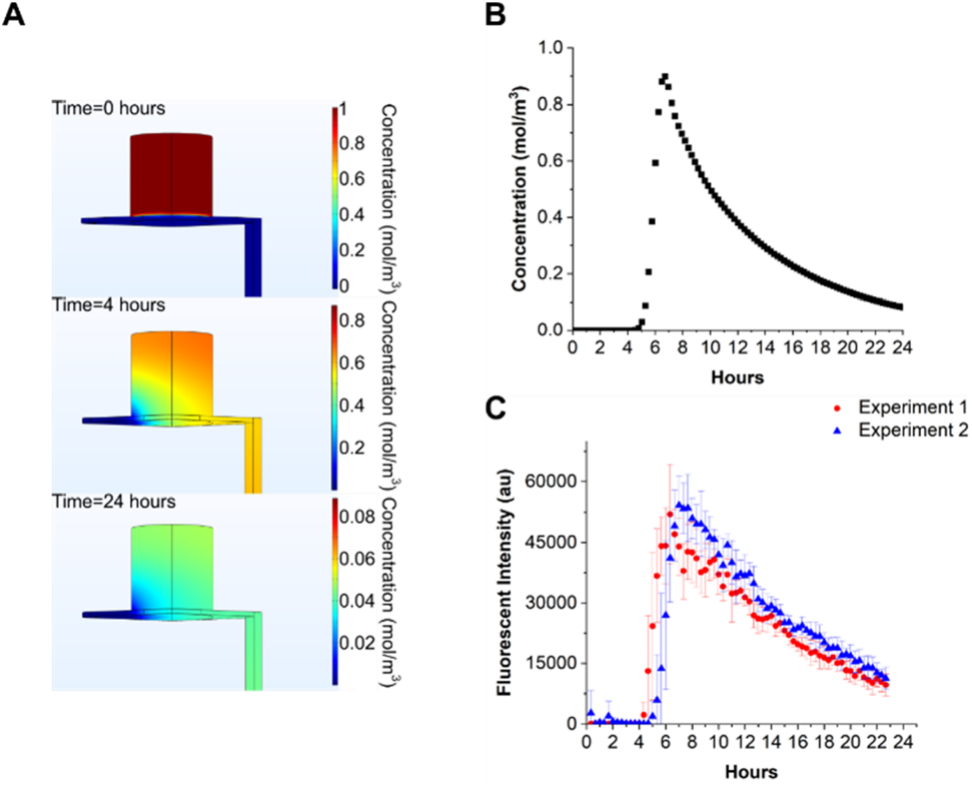
(A) COMSOL simulation of solute transport in the system. The initial solute (FITC) concentration in the apical compartment was set to 1 mol m^−3^, with a basolateral flow of 30 μL h^−1^. Solute diffusion coefficient was set to 4 × 10^−10^ m^2^ s^−1^. The nanoporous membrane between basolateral and apical compartments was modelled as a porous matrix with a porosity of 0.126. Concentration in the apical compartment decreases with time, as the solute diffuses across the membrane into the basolateral flow. After 24 hours, approximately 5% of the initial concentration is present in the apical compartment. (B) Calculated time-dependent concentration of the solute at the outlet showing a peak at 6 hours. (C) Experimentally measured droplet fluorescent intensity *vs.* time. Two separate experiments for FITC diffusing across the microfluidic chip membrane. Each point is the average fluorescent intensity measured from 8 independent microfluidic chips ± standard deviation.

To estimate the time evolution of solutes in the device, COMSOL simulations were performed. The microfluidic chip was modelled as a fixed volume container with an outlet tube of 1 mm internal diameter, 10 cm long. The nanoporous membrane in the microfluidic chip, onto which cells are seeded was modelled as a porous matrix, with a porosity of 12.6% and 12 μm thickness. The porosity value was calculated from the physical properties of a nanoporous membrane with circular pores of 0.4 μm diameter at a density of 10^8^ pores per cm^2^. A fixed flow rate of 30 μL h^−1^ was set at the inlet of the chip. The solute in the apical chamber was set to 1 mM, the volume to 100 μL, and the concentration calculated at the outlet as a function of time. The diffusion coefficient of the solute (FITC) was set to 4 × 10^−10^ m^2^ s^−1^.^30^ The flow profile in the chip and tubing was calculated from the Navier–Stokes equation, and mass transport calculated from the convection–diffusion equation, using the calculated velocity field values. The simulation results are shown in Fig. 4 and demonstrate how the solute in the apical compartment appears in the droplets after 4–4.5 hours with a peak concentration at 6–7 hours, followed by a decay in concentration over time with a time constant of 7.6 hours (Fig. 4B). The simulations show that the solute decay profile and peak concentration did not vary considerably between the end of the microfluidic chip and the sampling location (see ESI Fig. 3†).

To experimentally measure the diffusion of a solute through the nanoporous membrane, water was pumped through all 8 microfluidic chips for 24 hours. 100 μL of FITC solution (2 mg mL^−1^) was added to the apical compartment of each chip and droplets generated every 20 minutes. After manually dispensing droplets into 384 well plates the fluorescence was measured with a plate reader (Promega Gloxmax, 520_EX_/580–640_EM_). The results of two independent experimental repeats are shown in Fig. 4C. The experimental data correlates with simulation, where FITC diffused through the nanoporous membrane and appeared approximately 4–5 hours after the experiment started, peaking at 6–8 hours. The decay in FITC concentration is consistent with the reduction in concentration in each chip with time. The mean time constants for decay rate were 10.36 ± 0.05 hours, higher compared to the simulated value. The experiment was performed twice and it was ascertained that the main source of error for both experiments was due to manual dispensing of droplets into the 384 well plate.

**Fig. 5:**
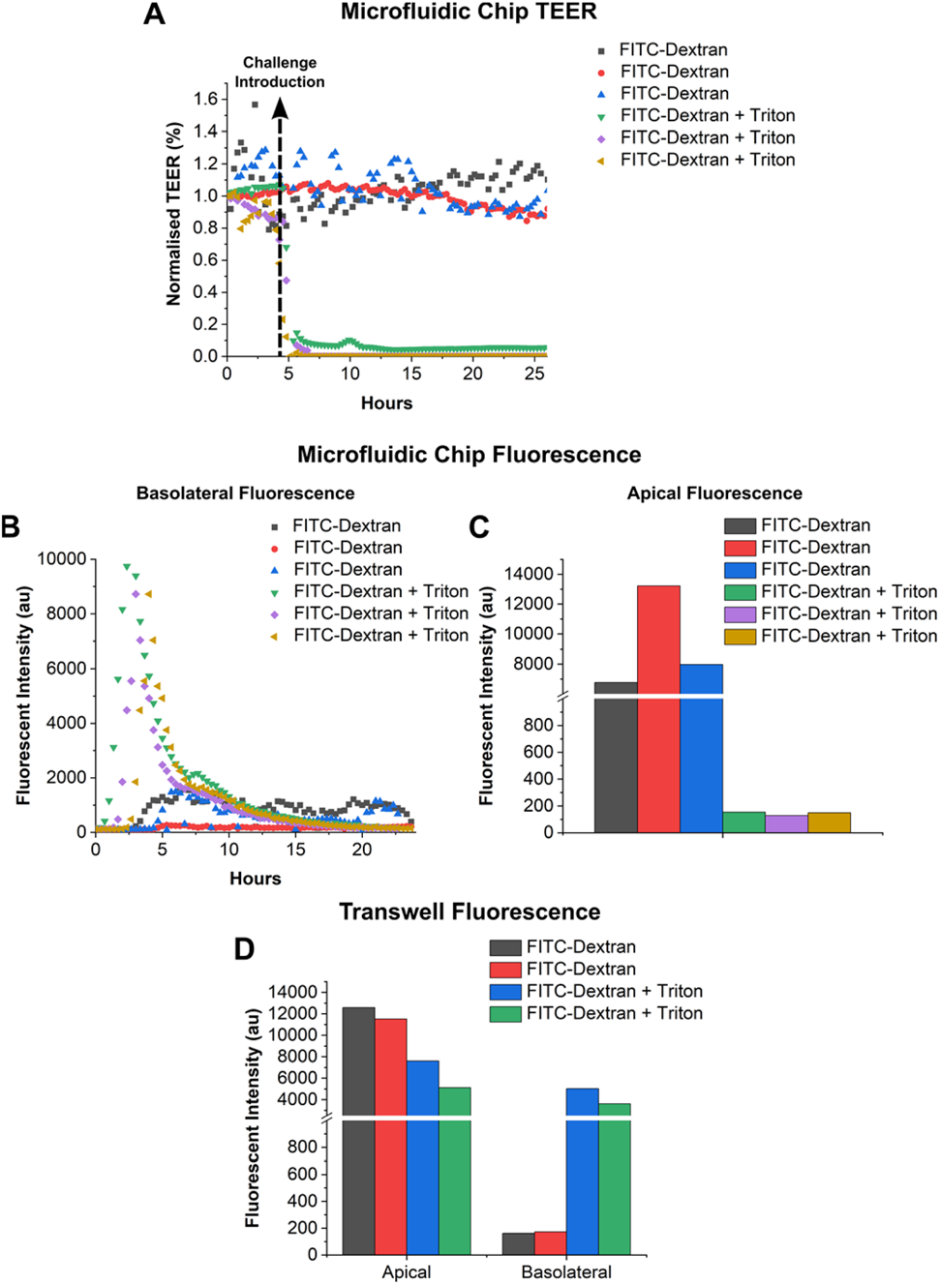
(A) Normalised TEER for cells grown on the OoC platform with FITC-Dextran 2 mg mL^−1^ added to the apical compartment at *t* = 4 hours. Data is without (black, red and blue) or with addition of TX-100 (green, purple and yellow). (B) Fluorescence intensity of single droplets *vs.* time after addition of TX-100 showing diffusion of FITC-Dextran into the basolateral flow. (C) Fluorescent intensity from a 10 μL sample of the apical medium from each microfluidic chip 24 hours post-challenge. (D) Apical and basolateral fluorescence from a 10 μL sample of media taken from cells grown on Transwell inserts after 24 hours of FITC-Dextran exposure with (blue and green) or without (black and red) TX-100. Data is from one chip from 3 separate experiments.

Having demonstrated that the system performs as expected it was then used to measure the macromolecular permeability of a high molecular weight sugar (FITC-Dextran) across an epithelial cell barrier. Human bronchial epithelial cells (16HBE14o-) were grown for 4 days with TEER measured continuously to determine the ionic permeability.^29^ Following barrier formation, FITC-Dextran (10 kDa) was added to the apical side of 6 separate chips, to a final concentration of 2 mg mL^−1^. In 3 out of 6 chips a solution of TX-100 (apical concentration of 1% v/v) was mixed with the FITC-Dextran to disrupt the tight junctions. After addition of TX-100, droplets were generated every 20 minutes for 24 hours and at the end of the experiment droplet fluorescence was measured, along with 10 μL from the apical compartment. Cells were also cultured on Transwells (without flow) and treated in the same way after 4 days of cell growth. 24 hours after addition of FITC-Dextran and TX-100, 10 μL of the apical and basolateral solution from each Transwell was dispensed into a 384 well plate for analysis. The results are shown in Fig. 5.

An intact epithelial cell barrier inhibits diffusion of FITC-Dextran between the apical and basolateral compartments. Addition of Triton led to cell membrane permeabilization and rapid epithelial cell barrier disruption (as shown by the sharp decrease in TEER in Fig. 5A).^31–33^ Droplets collected from chips without TX-100 treatment show a constant low-level flux of FITC-Dextran across the barrier. The fluorescence rises above baseline 3–4 hours post addition, remaining stable over the following 24 hours indicating a low flux of the FITC-Dextran across the cell barrier. For cells exposed to TX-100, the fluorescence intensity of the droplets peaked at 3–4 hours post challenge, then decayed as the FITC-Dextran slowly diffused through the nanoporous membrane (Fig. 5B) as seen in the data shown in Fig. 4C. These data correlate with the Transwell end point measurements. Transwells treated with Triton had equal concentration of FITC-Dextran on either side of the membrane (Fig. 5D) as expected, since the solute is not depleted by mass transport (no flow). In the static system, diffusion drives the system to equilibrium with both compartments having approximately the same concentration of solute after 48 hours, as predicted by simulation.

## Conclusions

We have described a system capable of collecting and compartmentalising secretions in eluent from an organ-on-chip platform. The system uses a simple droplet microfluidic generator which can be easily attached to existing OoC platforms, providing temporal analysis of molecular permeability or cytokine production. This increases the temporal resolution compared to current methods such fraction collectors or manual collection.

The system comprises a PDMS droplet generator and a PMMA pressure controller, and can generate droplets in 8 different microfluidic channels simultaneously using two pressure supplies. The system was coupled to an OoC platform with a flow rate of 30 μL h^−1^, generating droplets every 20 minutes, giving an average droplet volume of 9.47 ± 0.6 μL for all 8 channels. Diffusion and compartmentalisation of a fluorescent compound (FITC) was measured and compared to simulations, showing good correlation. The molecular permeability of human bronchial epithelial cells to FITC-Dextran was measured showing a clear difference between cells with and without Triton X-100. Cell membrane disruption caused the Triton X-100 led to a peak in fluorescence 3–4 hours after stimulation. In the absence of Trion, a constant low-level of FITC-Dextran was measured. This data was consistent with Transwells end point measurements.

The system is capable of compartmentalising analytes from 8 parallel microfluidic channels simultaneously in an automated manner and provides an easy way of collecting temporal data of for example drug permeability or cytokine production.

## Data availability

All data supporting this study is openly available from the University of Southampton repository at https://doi.org/10.5258/SOTON/D2378.

## Author contributions

Joao Fernandes was involved in the conceptualisation, data curation, formal analysis, investigation, methodology, resources, software, validation and writing – original draft; Nikita Karra was involved in the methodology (supporting) and writing – review and editing; Emily J. Swindle was involved in conceptualization, supervision, funding acquisition, project administration, resources and writing – review and editing; Hywel Morgan was involved in conceptualization, supervision, funding acquisition, project administration, resources and writing – review and editing.

## Conflicts of interest

There are no conflicts to declare.

## Supplementary Material

RA-013-D3RA00470H-s001

## References

[cit1] Apostolou A. (2021). *et al.*, A Novel Microphysiological Colon Platform to Decipher Mechanisms Driving Human Intestinal Permeability. Cmgh.

[cit2] Pediaditakis I. (2021). *et al.*, Modeling alpha-synuclein pathology in a human brain-chip to assess blood–brain barrier disruption. Nat. Commun..

[cit3] Kimura H., Yamamoto T., Sakai H., Sakai Y., Fujii T. (2008). An integrated microfluidic system for long-term perfusion culture and on-line monitoring of intestinal tissue models. Lab Chip.

[cit4] Rogers M. T. (2021). *et al.*, A high-throughput microfluidic bilayer co-culture platform to study endothelial–pericyte interactions. Sci. Rep..

[cit5] Eickenberg B. (2013). *et al.*, Lab-on-a-chip magneto-immunoassays: How to ensure contact between superparamagnetic beads and the sensor surface. Biosensors.

[cit6] Sista R. , Florida State University Libraries Development of a Digital Microfluidic Lab-on-a-Chip for Automated Immunoassay with Magnetically Responsive Beads, 2007

[cit7] Pham N. M. (2019). *et al.*, Immuno-gold silver staining assays on capillary-driven microfluidics for the detection of malaria antigens. Biomed. Microdevices.

[cit8] Mahato K. , *et al.*, Electrochemical immunosensors: Fundamentals and applications in clinical diagnostics, Handbook of Immunoassay Technologies: Approaches, Performances, and Applications, Elsevier Inc., 2018, 10.1016/B978-0-12-811762-0.00014-1

[cit9] Warsinke A., Benkert A., Scheller F. W. (2000). Electrochemical immunoassays. Fresenius' J. Anal. Chem..

[cit10] Fowler J. M. , Wong D. K. Y., Brian Halsall H. and Heineman W. R., Recent developments in electrochemical immunoassays and immunosensors, Electrochemical Sensors, Biosensors and their Biomedical Applications, Elsevier Inc., 2008, 10.1016/B978-012373738-0.50007-6

[cit11] Yakoh A., Chaiyo S., Siangproh W., Chailapakul O. (2019). 3D Capillary-Driven Paper-Based Sequential Microfluidic Device for Electrochemical Sensing Applications. ACS Sensors.

[cit12] Kerns S. J. (2021). *et al.*, Human immunocompetent organ-on-chip platforms allow safety profiling of tumor-targeted t-cell bispecific antibodies. Elife.

[cit13] Si L. (2021). *et al.*, A human-airway-on-a-chip for the rapid identification of candidate antiviral therapeutics and prophylactics. Nat. Biomed. Eng..

[cit14] Zhu Y. (2021). *et al.*, State of the art in integrated biosensors for organ-on-a-chip applications. Curr. Opin. Biomed. Eng..

[cit15] Shin S. R. (2017). *et al.*, Label-Free and Regenerative Electrochemical Microfluidic Biosensors for Continual Monitoring of Cell Secretomes. Adv. Sci..

[cit16] Aleman J., Kilic T., Mille L. S., Shin S. R., Zhang Y. S. (2021). Microfluidic integration of regeneratable electrochemical affinity-based biosensors for continual monitoring of organ-on-a-chip devices. Nat. Protoc..

[cit17] Ryon Shin S. (2016). *et al.*, Aptamer-Based Microfluidic Electrochemical Biosensor for Monitoring Cell-Secreted Trace Cardiac Biomarkers HHS Public Access. Anal. Chem..

[cit18] Lee J. (2021). *et al.*, A Heart-Breast Cancer-on-a-Chip Platform for Disease Modeling and Monitoring of Cardiotoxicity Induced by Cancer Chemotherapy. Small.

[cit19] Hu J., Li X., Judd R. L., Easley C. J. (2020). Rapid lipolytic oscillations in: ex vivo adipose tissue explants revealed through microfluidic droplet sampling at high temporal resolution. Lab Chip.

[cit20] Adams A. G., Bulusu R. K. M., Mukhitov N., Mendoza-Cortes J. L., Roper M. G. (2019). Online Measurement of Glucose Consumption from HepG2 Cells Using an Integrated Bioreactor and Enzymatic Assay. Anal. Chem..

[cit21] Blume C. (2015). *et al.*, Temporal monitoring of differentiated human airway epithelial cells using microfluidics. PLoS One.

[cit22] Nawroth J. C. (2020). *et al.*, A microengineered airway lung chip models key features of viral-induced exacerbation of asthma. Am. J. Respir. Cell Mol. Biol..

[cit23] Chrobak K. M., Potter D. R., Tien J. (2006). Formation of perfused, functional microvascular tubes in vitro. Microvasc. Res..

[cit24] Villenave R. (2017). *et al.*, Human gut-on-a-chip supports polarized infection of coxsackie B1 virus in vitro. PLoS One.

[cit25] Nightingale A. M. (2019). *et al.*, Monitoring biomolecule concentrations in tissue using a wearable droplet microfluidic-based sensor. Nat. Commun..

[cit26] Kennedy R. T. (2013). Emerging trends in *in vivo* neurochemical monitoring by microdialysis. Curr. Opin. Chem. Biol..

[cit27] Evans G. W. H. (2021). *et al.*, A portable droplet microfluidic device for cortisol measurements using a competitive heterogeneous assay. Analyst.

[cit28] Cedillo-Alcantar D. F., Han Y. D., Choi J., Garcia-Cordero J. L., Revzin A. (2019). Automated Droplet-Based Microfluidic Platform for Multiplexed Analysis of Biochemical Markers in Small Volumes. Anal. Chem..

[cit29] Fernandes J. (2022). *et al.*, Real-Time Monitoring of Epithelial Barrier Function by Impedance Spectroscopy in a Microfluidic Platform. Lab Chip.

[cit30] Casalini T., Salvalaglio M., Perale G., Masi M., Cavallotti C. (2011). Diffusion and aggregation of sodium fluorescein in aqueous solutions. J. Phys. Chem. B.

[cit31] Mermoud Y., Felder M., Stucki J. D., Stucki a. O., Guenat O. T. (2018). Microimpedance tomography system to monitor cell activity and membrane movements in a breathing lung-on-chip. Sens. Actuators, B.

[cit32] Sun T., Tsuda S., Zauner K. P., Morgan H. (2010). On-chip electrical impedance tomography for imaging biological cells. Biosens. Bioelectron..

[cit33] Koley D., Bard A. J. (2010). Triton X-100 concentration effects on membrane permeability of a single HeLa cell by scanning electrochemical microscopy (SECM). Proc. Natl. Acad. Sci. U. S. A..

