# Supplementary material for: Droplet fluidics for time-dependent analysis of barrier permeability in an epithelial barrier on chip system
Source: RSC Adv. 2023 May 11;13(21):14494–500. doi: 10.1039/d3ra00470h (PMC10173818; doi:10.1039/d3ra00470h)
Supplement: RA-013-D3RA00470H-s001 [file RA-013-D3RA00470H-s001.pdf]

Electronic Supplementary Material (ESI) for RSC Advances.
This journal is © The Royal Society of Chemistry 2023

System Fabrication

The pressure controller is made of 3 layers of PMMA. The top and bottom layers provide structural composition to the device; the top layer has the pressure inlets and the bottom layer the pressure outlets. The middle layer contains the microchannels which route air pressures from inlet (500 or -500 mbar) to outlet via solenoid valves. Actuation of the solenoid valves leads to pressurization of a specific outlet (located at the middle of the device). Outlets are subsequently connected to the pressure channels in the droplet generator device, controlling the PDMS microvalves there present (Fig. 2B).

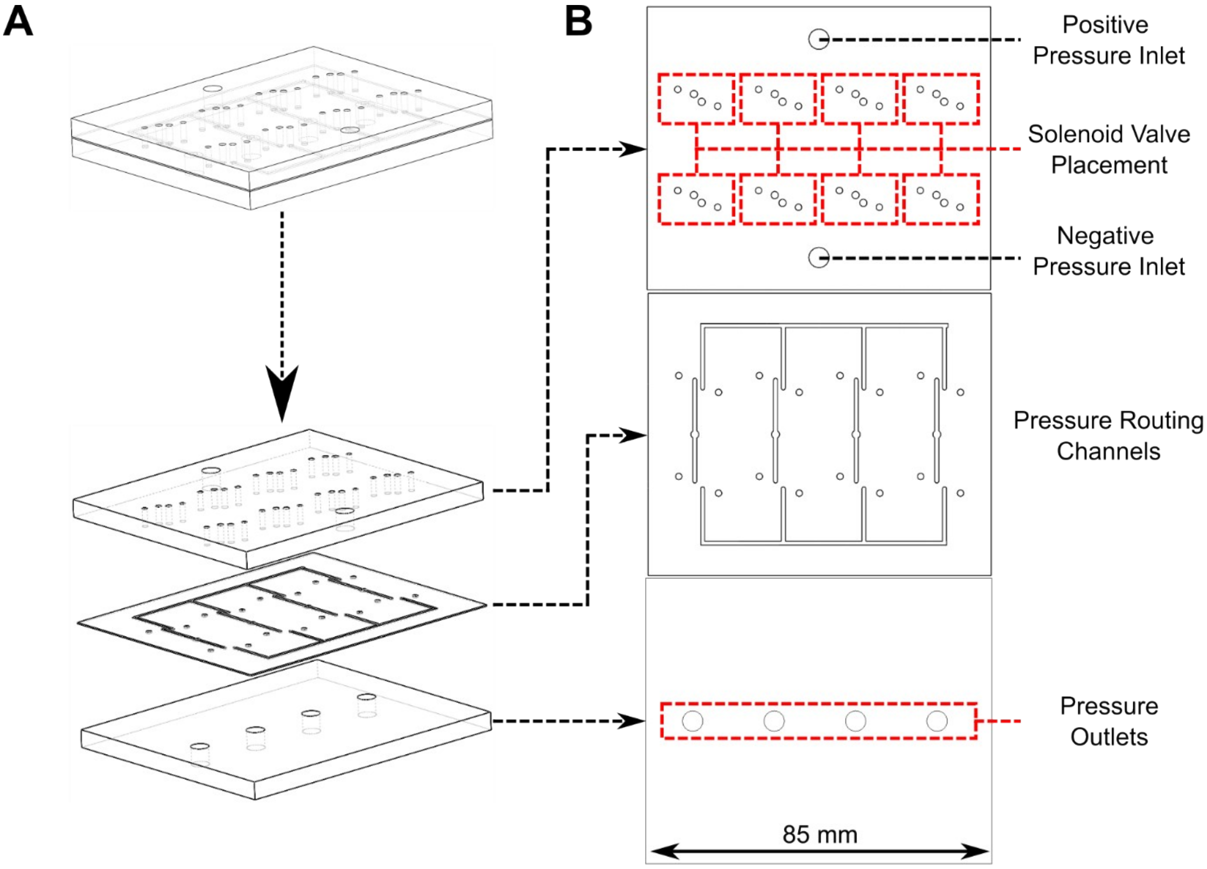

Fig. 1: (A) 3D Schematic and exploded view of the pressure controller. (B) Top-down view of each of the 3 layers that compose the peristaltic controller and location of inlets, outlets, and solenoid valves for pressure routing.

The droplet generator is made of 3 x PDMS layers (Fig 4). The top and bottom layers have microchannels for pressurised air and microfluidics respectively. The middle layer is a thin PDMS membrane that is deflected the pressure and is used to control both oil and cell media. The top layer inlets are connected to the pressure controller outlets and are provided with positive or negative pressures. The 4 pressure channels are used for two purposes, a peristaltic pump, responsible for automated and time dependent oil flow, and a microvalve, inhibiting backflow of oil into the platform during peristaltic actuation. All 8 microfluidic channels are simultaneously controlled by the 4 pressure inlets. Oil and cell media droplets are sequentially stored in PTFE tubing attached to the droplet generator outlet.

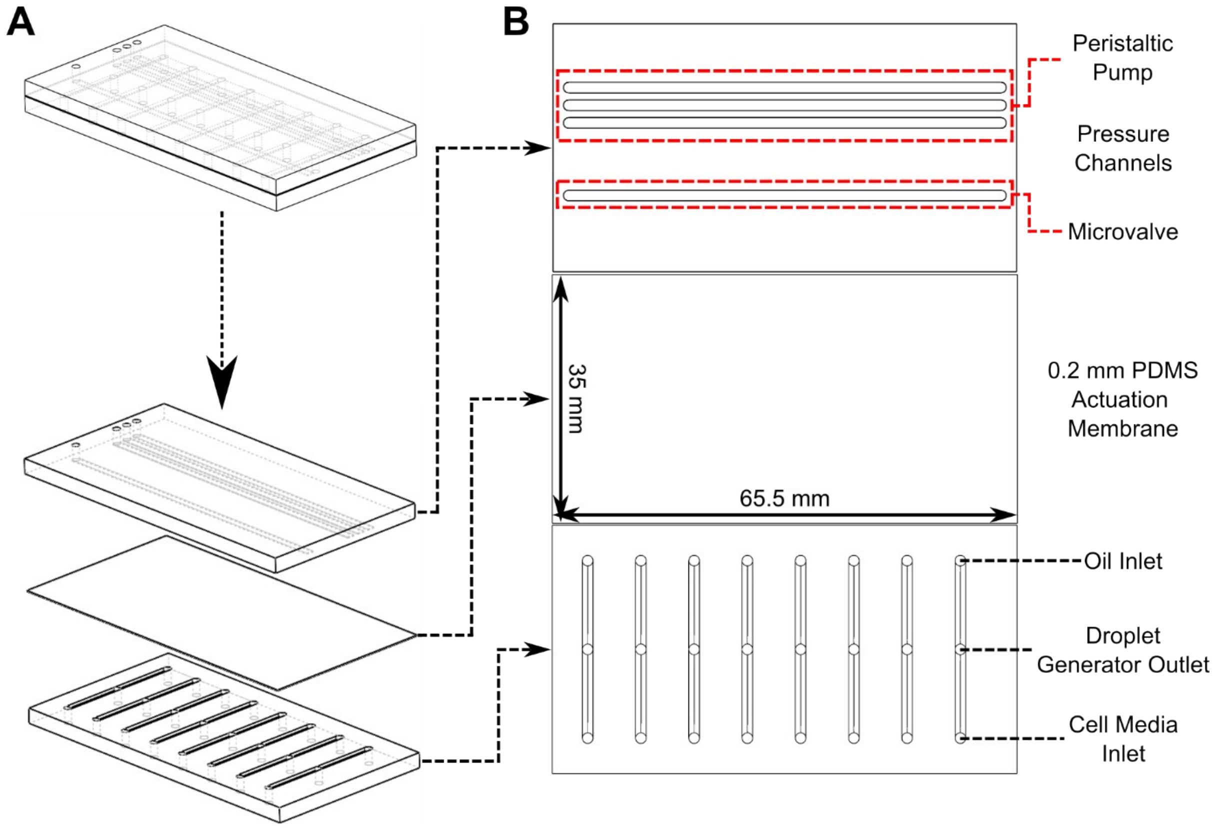

Fig. 2: (A) 3D Schematic and exploded view of the droplet generator. (B) Top-down view of each of the 3 layers that compose the droplet generator and location of inlets and outlets for air pressure (top layer) or liquids (bottom layer).

Solute profile before and after sampling point

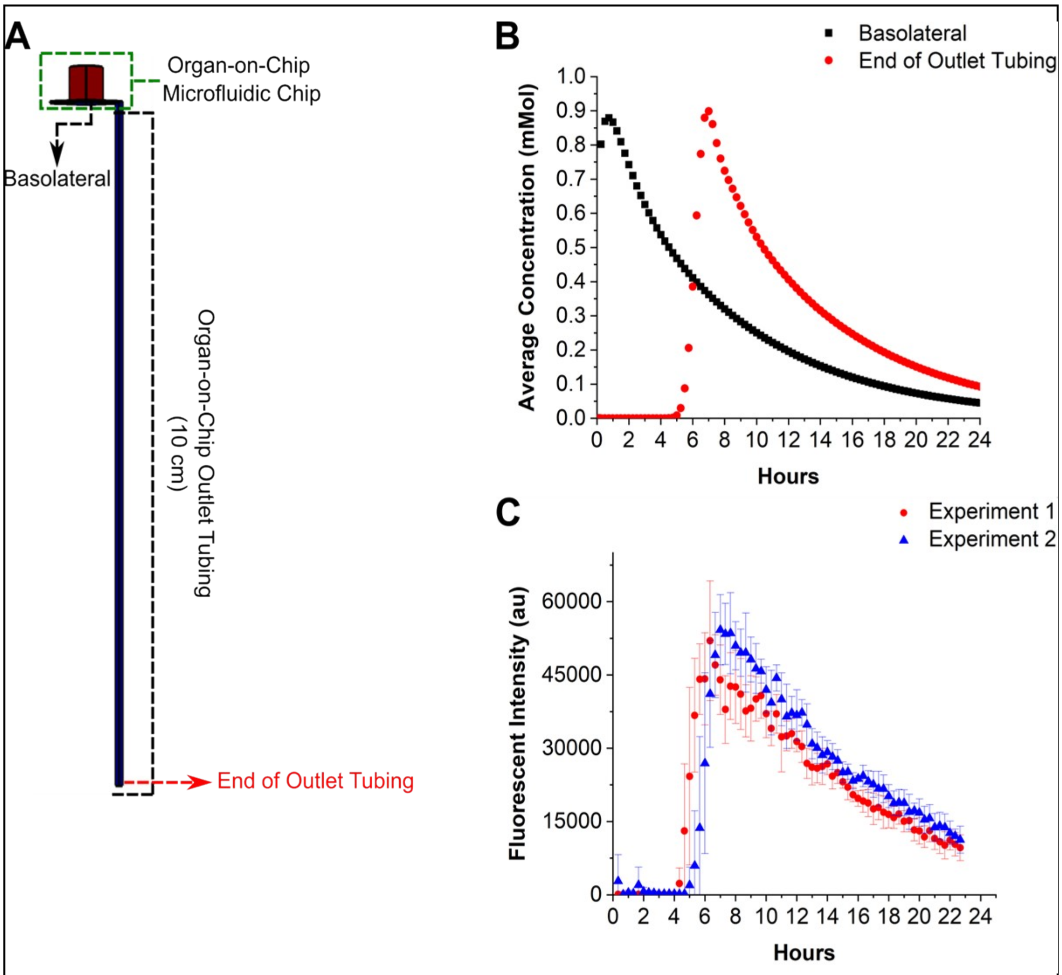

Figure 3 COMSOL diagram (A) displaying the microfluidic chip and locations were solute concentration was measured and compared. Simulated data of average solute concentration present in the basolateral compartment and at the end of the outlet tubing of the OoC platform (B), to which the droplet generator will be attached. Diffusion of FITC through the OoC porous support using the droplet generator over the course of 24 hours (C), where each data point represents a different collected droplet and each coloured line a different experimental repeat. Error bars in C correspond to the standard deviation across all 6 microfluidic chips, while each point relates to the average.
